# SEOM clinical practice guideline: management and prevention of febrile neutropenia in adults with solid tumors (2018)

**DOI:** 10.1007/s12094-018-1983-4

**Published:** 2018-11-23

**Authors:** A. Carmona-Bayonas, P. Jimenez-Fonseca, E. M. de Castro, E. Mata, M. Biosca, A. Custodio, J. Espinosa, E. G. Vázquez, F. Henao, F. Ayala de la Peña

**Affiliations:** 1 Hematology and Medical Oncology Department, Hospital Universitario Morales Meseguer, IMIB, Avenida Marqués de los Vélez, 30008 Murcia, Spain; 2 Medical Oncology Department, Hospital Universitario Central de Asturias, Oviedo, Spain; 3 Medical Oncology Department, Hospital Universitario Marqués de Valdecilla, Santander, Spain; 4 Medical Oncology Department, Complejo Hospitalario de Navarra, Pamplona, Spain; 5 Medical Oncology Department, Hospital Vall D’Hebron, Barcelona, Spain; 6 Medical Oncology Department, Hospital Universitario La Paz, Madrid, Spain; 7 CIBERONC (CB16/12/00398), Madrid, Spain; 8 Medical Oncology Department, Hospital General de Ciudad Real, Ciudad Real, Spain; 9 Infectious Disease Department, Hospital Universitario Virgen de la Arrixaca, Murcia, Spain; 10 Departamento de Medicina, Facultad de Medicina, Universidad de Murcia, Murcia, Spain; 11 Medical Oncology Department, Complejo Hospitalario Regional Virgen Macarena, Seville, Spain

**Keywords:** CISNE score, Febrile neutropenia, SEOM, Clinical practice guideline

## Abstract

Febrile neutropenia (FN) is a common dose-limiting toxicity of chemotherapy, with a profound impact on the evolution of patients with cancer, due to the potential development of serious complications, mortality, delays, and decrease in treatment intensity. This article seeks to present an updated clinical guideline, with recommendations regarding the diagnosis, prevention, and treatment of febrile neutropenia in adults with solid tumors. The aspects covered include how to properly approach the risk of microbial resistances, epidemiological aspects, considerations about the initial empirical approach adapted to the risk, special situations, and prevention of complications. A decision-making algorithm is included for use in the emergency department based on a new, validated tool, the Clinical Index of Stable Febrile Neutropenia, which can be used in patients with solid tumors who appear stable in the initial phase of neutropenic infections, and can help detect those at high risk for complications in whom early discharge must be avoided.

## Introduction

Febrile neutropenia (FN) is defined as the appearance of fever (temperature > 38 °C) or clinical signs of sepsis, in a patient with a neutrophil count of < 500/mm^3^ or < 1000/mm^3^ pre-nadir [1, 2]. It is a common iatrogenic complication, varying in incidence (2–50%), depending on patient-related risk factors, neoplasm, type of chemotherapy, and genetic susceptibility [3–5]. While most patients experience mild episodes, the rate of serious complications (25–30%) and mortality (9–12%) remains elevated in high-risk groups [6]. The physiopathology of neutropenic sepsis covers the disruption of mucosal barriers, the architecture of the gastrointestinal lymphatic system, and the dissemination of bacterial inoculates throughout the bloodstream. Emergency (< 1 h) initiation of broad-spectrum antimicrobial therapy is indispensable to preserve clinical stability.

In addition to its pharmacoeconomic impact [7, 8], neutropenia generates delays and dose reductions [9], which decreases the efficacy of chemotherapy [10, 11]. Primary prophylaxis of FN may be necessary for the most aggressive antineoplastic regimens or, in certain contexts, in subgroups of more vulnerable patients (e.g., the elderly) [12, 13].

The aim of this guideline, developed by the Spanish Society of Medical Oncology (SEOM, for its acronym in Spanish), is to set for the updated directives for the evidence-based diagnosis and treatment of FN in patients with solid tumors.

## Methods

These guidelines have been developed by SEOM with the consensus of a panel of ten experts in the management of immunocompromised patients with solid tumors. The assignment of a level of evidence and grade of recommendation for each statement follows the Infectious Diseases Society of America-US Public Health Service Grading System for Ranking Recommendations in Clinical Guidelines. A full list of recommendations for febrile neutropenia is provided in Table 1.

**Table 1:** General recommendations and levels of evidence

Diagnostic/treatment recommendation	Level, Grade
*Initial clinical evaluation*	
The initial assessment should include the clinical history, physical examination, complete blood count, and basic biochemistry, and chest Rx	III, B
Two pairs of blood cultures and microbiological specimens, as clinically indicated, should be obtained prior to initiating empirical antibiotic therapy	III, A
Initial evaluation may include procalcitonin levels for the diagnosis of presumed bacterial infection and prognostic stratification	II, B
Consider a more comprehensive microbiological study in patients with clinical suspicion or history of specific infections, or with severe immunosuppression	III, B
Perform a computerized tomography of the chest in patients with clinically relevant respiratory symptoms and inconclusive chest Rx, or in patients with persistent fever (72 h or more) and risk factors for complications	II, B
*Initial prognostic evaluation*	
An initial prognostic evaluation according to clinical criteria is recommended to identify unstable patients or those at risk of severe complications	III, B
The application of the CISNE score is recommended in patients with apparent clinical stability and solid tumors on moderate-intensity chemotherapy	II, B
*Initial empirical treatment*	
Empirical antibiotic therapy should be initiated within the first hour of arrival at the ED (after sampling for cultures)	II, A
Initial parenteral antibiotherapy should include a beta-lactam with activity against *Pseudomonas* spp. (piperacillin/tazobactam, meropenem, imipenem–cilastatin, cefepime)	I, A
In high-risk patients meeting criteria for severity, initial parenteral antibiotherapy should include a beta-lactam with activity against *Pseudomonas* spp. (preferably a carbapenem) in combination with amikacin	II, A
In high-risk patients with suspicion of catheter-related infection or infection with a skin focus, pneumonia, or hemodynamic instability, it is recommended to associate vancomycin, linezolid (of choice if the focus is either pulmonary or cutaneous, but not recommended in catheter-related infections), or daptomycin (of choice in severe patients with quick SOFA ≥ 2 points and suspicion of cutaneous or catheter focus) to initial antibiotherapy. Tigecycline should be used only as a last option	II, A
In high-risk patients with enterocolitis or perirrectal infection, metronidazole should be associated to a beta-lactam with antipseudomonal activity	II, A
In patients who are allergic to penicillin, we recommend aztreonam and vancomycin, with association of amikacin in situations of severity or *Pseudomonas aeruginosa* infection	II, B
In low-risk patients without prior prophylaxis with fluoroquinolones, after parenteral administration of the first doses, oral treatment can be undertaken with the combination of amoxicillin–clavulanic and levofloxacin or ciprofloxacin	II, B
In low-risk patients, oral fluoroquinolones in monotherapy should not be used	II, D
Therapeutic use of G-CSF is recommended in patients at high risk for infectious complications, with neutropenia < 100 neutrophils/mm^3^ or in the presence of risk factors (age > 65, clinical instability, widespread infection, or severe complication)	I, A
*Treatment in special situations*	
Antimicrobial treatment should be adapted to the isolates and patterns of resistance (detailed in Table 3)	II, A
Empirical antifungal treatment should be considered if the fever persists after 5–7 days and microbiological diagnoses are negative	I, A
If the fever persists after 5–7 days and microbiological diagnoses are negative, studies aimed at ruling out invasive fungal infection should be performed (determination of blood galactomannan titers, studies guided by clinical suspicion)	III, A
*Hygiene-dietary measures*	
Prescription of a low-bacteria diet may be considered in patients with febrile neutropenia	III, C
Strict hand-washing protocols should be followed before and after contact with a patient with febrile neutropenia (FN)	I, A
Oral and skin hygiene should be optimized in patients with FN	II, A
Patients with resistant bacterial infections (methicillin-resistant *Staphylococcus aureus*, vancomycin-resistant *Enterococcus spp,* carbapenemase-resistant *Enterococcus spp*, or ESBL-producing *Enterobacteriaceae*, non-fermenting Gram-negative bacilli, such as *Pseudomonas aeruginosa* or *Acznetobacter baumannii*, with a multi-resistant pattern) should be isolated	II, A
Reverse isolation is recommended only in patients treated with chemotherapy associated with profound, prolonged neutropenia	II, A
*Follow-up and adjusting treatment*	
Daily clinical and analytical follow-up, with blood cultures every 48 h (and samples from other sites if applicable) in situations of persistent fever are recommended in patients with FN	III, A
Prevention and treatment of associated complications (thrombosis, cytopenias, mucositis) should be performed in patients with FN	III, A
Antibiotic treatment should be adapted to clinical suspicion, the infectious focus, and microbiological isolate in cases of persistence of fever and/or identification of the infectious focus	I, A
Specific empirical treatment against Gram-positive germs should be withdrawn if suspicion is not confirmed within 48 h of initiation	II, B
Antibiotic coverage should be extended to Gram-negative bacilli, Gram-positive bacterium, and anaerobes in patients whose fever persists for more than 48 h associated with hemodynamic instability or clinical progression	I, A
Consultation with a specialist in infectious disease is recommended in bacteremias due to *S. aureus*, high-risk situations, multi-resistant germs, atypical sites or treatments, or HIV infection	III, C
Empirical antibiotic treatment should be maintained for at least 7 days in low-risk FN without a clinical or microbiological focal site	III, A
Consider switching to oral antibiotic treatment in patients with low risk for FN without clinical or microbiological focal site with neutrophil count exceeding 500/mm3 and without fever for at least 72 h	III, A
Antibiotic spectrum should be restricted according to infection focal site, severity, and the antibiogram of microorganisms identified as causing the infection after febrile neutropenia have resolved	II, A
Maintenance of antibiotherapy should be adjusted at least to the recommended duration for the underlying infection	III, B
Antibiotic treatment should last for at least 10–14 days in infections of the skin and soft tissue, pneumonias, and urinary tract infections	II, B
*Hospital or ambulatory treatment*	
Patients meeting severity criteria should be treated as inpatients	II, A
Patients not meeting severity criteria and who appear to be clinically stable and present high-risk CISNE scores (≥ 3 points) should be treated as inpatients	II, A
Patients not meeting severity criteria and who appear to be clinically stable and present high-risk CISNE scores (CISNE 3 or CISNE 2 with additional risk factors) should not be discharged from the hospital until clinical stability and negative results on blood culture are confirmed	II, B
Early discharge or ambulatory treatment with close follow-up may be considered in clinically stable patients not meeting criteria for severity, who exhibit low or intermediate risk scores on the CISNE model (CISNE 0–1) and who also meet the criteria for proximity, support, accessibility, and compliance with treatment and follow-up. However, CISNE does not specifically seek to select candidates for outpatient management, and standard hospitalization is also acceptable for these patients	III, B
In low-risk patients for whom ambulatory treatment has been decided on, the first dose of antibiotic should be administered via IV within the first hour and the patient should remain in observation at the hospital for at least 4 h	III, B
Close supervision should be maintained of low-risk patients for whom ambulatory treatment has been decided on	III, B
In low-risk patients for whom ambulatory treatment has been decided on, detailed oral and written information should be provided regarding warning signs and symptoms, and contact data	III, B
Hospital readmission is recommended in low-risk patients for whom ambulatory treatment has been decided on who continue to have febrile neutropenia after 48–72 h of empirical treatment or/and who present new information about infection, oral intolerance, or for whom a change of antibiotic is indicated	III, B
*Prevention of febrile neutropenia*	
Primary prophylaxis with filgrastim, pegfilgrastim, or biosimilar drugs is recommended when the probability of FN exceeds 20%	I, A
The indication for primary prophylaxis with filgrastim, pegfilgrastim, or biosimilar drugs in patients with intermediate risk for neutropenic fever (10–20%) should be individualized. The qualitative consequences of neutropenic infections, and not just their probabilities, must be considered	II, A
Secondary prophylaxis with filgrastim, pegfilgrastim, or biosimilar drugs is recommended in patients with a previous episode of FN in whom delaying or lowering the dose can negatively impact survival	I, A
As a general recommendation, antibiotic prophylaxis for FN should not be administered to patients undergoing mild–moderate-intensity chemotherapy	I, A
In patients undergoing mild–moderate-intensity chemotherapy, antibiotic prophylaxis for FN may considered in exceptional cases with very high risk of infection	III, B
Prophylaxis should be assessed for patients with specific risks for *P. jirovecii*, tuberculosis, or hepatitis	II, A
Influenza vaccination is recommended in patients with solid tumors with active disease or/and undergoing chemotherapy	II, A
Vaccination against pneumococcus is recommended in patients with solid tumors with active disease or/and undergoing chemotherapy	III, A

## Etiology and antimicrobial susceptibility patterns

In patients with solid tumors, FN has an identifiable clinical focus in 65% of the episodes; however, microbiological documentation is only possible in 20–30% of the cases and blood cultures are positive in 10–25% [14, 15]. Etiology is usually bacterial, due to both Gram-negative bacilli, as well as Gram-positive cocci (approximate ratio of 3:2). Selective pressures that favor Gram-positive cocci infections in hematological patients (central venous catheter [CVC], prophylaxis with quinolones, mucositis grade 3–4, etc.) are less intense in patients with solid cancer [15, 16]. Anaerobic microorganisms and polymicrobial infections are uncommon, but can occur in special situations (e.g., abscesses, enteritis, etc.).

In recent years, there has been an increase in strains resistant to extended-spectrum β-lactamase (ESBL) or carbapenemases [17]. The risk of resistant microorganisms depends on previous colonization, invasive procedures, prior exposure to antibiotics, prior hospitalization, chronic comorbidity, and local pattern of resistances [II] [18–20]. The pattern of resistance recorded in Spain in 2015 for Gram positive is 10–25% to macrolides, in the case of pneumococcus; 25–50% to cloxacillin, in the case of *Staphylococcus aureus*; up to 90% for coagulase-negative staphylococci, and 1–5% resistance to vancomycin in the case of *Enterococcus faecium* [I, A] [21].

Invasive fungal infections (IFIs) are rare in patients with solid tumors (< 8%) [22]. Risk factors for IFI include prior use of antibiotics, having received multiple lines of chemotherapy, high-dose steroids (doses equivalent to or exceeding 20 mg/day of prednisone for 4 weeks or more), extensive mucositis, CVC, and prolonged neutropenia (> 7 days). *Candida albicans* is responsible for most candidemias, with a recent increase in infections due to fluconazole-resistant species (e.g., *Candida krusei* and *Candida glabrata*). Seasonal respiratory viruses are common in contact with infected individuals, but reactivation of other latent viruses or relevant pathogens, common in the context of acute leukemia or bone marrow transplant, is unusual in the case of solid tumors [II, C].

## Initial evaluation and risk stratification

The clinical history seeks to evaluate severity and clarify the clinical focus and epidemiological context (e.g., recent history of antibiotic use and the patient’s microbiological record). To identify the infectious focus, a systematic physical examination is essential, targeting potential foci of infection (CVC, skin, and soft tissues, perineal and perianal area, respiratory system, oral cavity, pharynx, sinus pressure points, abdominal examination; as well as neurological and eye fundus examination, if symptoms such as scotomas or blurry vision are present).

Before administering empirical antibiotics, two sets of samples for blood cultures from different anatomic sites (if patient has CVC, at least, one of the samples should be taken through the catheter) and microbiological samples should be taken, depending on the clinical orientation (e.g., urine, sputum, mucosal or skin lesions, feces, cerebrospinal fluid, urinary antigens for pneumococcus and/or *Legionella* spp., nasal swab for flu virus during flu season, etc.) [III, A]. A complete blood count and biochemistry is necessary with liver and kidney function tests, lactate, and a chest X-ray with two projections [III, B]. Procalcitonin (value > 0.5 ng/ml) may be more accurate and earlier than C-reactive protein (value ≥ 90 mg/dl) to guide the diagnosis of bacteremia, and in predicting severity and FN complications, although its benefit is unclear with respect to the information provided by clinical criteria [II, B] [23].

In patients with pulmonary infiltrates and more profound immunosuppression (e.g., steroids, prolonged neutropenia, use of immunosuppressants, etc.), early bronchoscopy with bronchoalveolar lavage may be indicated. A full microbiological study (e.g., virus, fungi, atypical bacteria) is indicated in selected patients with clinical suspicion, previous infections or immunosuppression in addition to neutropenia [III, B]. In individuals with respiratory symptoms and inconclusive chest X-ray, or with persistent fever ≥ 72 h and risk factors for complications, a chest computed tomography is useful to detect incipient pneumonias, with 87% sensitivity and a negative predictive value of 88%, surpassing chest Rx [24] [II, B]. Abdominal Rx has low sensitivity and specificity, and an abdominal CT is preferable in clinically suggestive cases and suspicion of abdominal focus (enterocolitis, typhlitis, etc.).

Severity is graded according to symptoms and signs, and risk assessment scores should only be applied when said signs and symptoms rule out clinical instability (see below) [III, B]. Most (3:1) individuals with FN and solid tumors display clinical stability in the first 3 h after diagnosis [25, 26]. High-risk patients or those who are potentially unstable are those with sepsis (defined as life-threatening acute organ dysfunction caused by the anomalous response to an infection, identifiable by an increase of 2 or more points on the quick SOFA scale), [27] septic shock, or severe documented infections (pneumonia, empyema, peritonitis, cellulitis > 4.5 cm, suspected typhlitis, enteritis grade 3–4, appendicitis, cholecystitis, or other complicated abdominal infections, meningitis, encephalitis, catheter infections, endocarditis, and pyelonephritis) [28, 29]. In these unstable patients, mortality due to sepsis is 18.1% versus 2.7% of patients without risk factors [29]. Infections entailing greater mortality are chronic obstructive pulmonary disease (COPD) exacerbations, pneumonia, bacteremia (specially those caused by Gram-negative bacilli), and invasive fungal infections [6, 14]. Tumor progression and lung cancer are associated with a worse prognosis [6, 30, 31]. Furthermore, other toxicities (e.g., mucositis, enteritis, thrombopenia) or coexisting complications (thromboembolic disease, need for transfusion, etc.) affect decision-making [25, 26, 32]. Table 2 is a complete list of clinical characteristics that define an episode of FN as being high risk.

**Table 2:** Risk factors for complications that contraindicate ambulatory management

Organ or system	Risk factor
Vascular	Syncope, systolic blood pressure < 90 mmHg, hypertensive crisis, arrhythmias, heart failure, clinically relevant bleeding, angina pectoris
Hematological	Other clinically relevant cytopenias (platelets < 50,000/μL, Hb < 8 g/dL), thromboembolic disease
Gastrointestinal	Oral intolerance, vomiting, diarrhea, pain abdominal, jaundice, alteration of liver function tests
Infectious	Sepsis or severe focus (pneumonia, extensive cellulitis, bacteremia, catheter, pyelonephritis, meningitis, cholecystitis, and other surgical infections), allergy to antibiotics, recent use of antibiotics
Neurological	Presence of focal neurological symptoms, suspicion of meningitis, acute confusional syndrome
Pulmonary	Abscesses, pneumothorax, pleural effusion, acute respiratory failure, pulmonary infiltrates, or cavitary nodules
Others	Acute kidney failure, dehydration, electrolyte alterations, other alterations of vital signs, other complications considered severe, pregnancy, fractures

Clinical assessment in subjects with inflammatory responses decreased by immunosuppression is complex, and some seemingly stable episodes remain at potential risk of complications, despite not showing evident criteria of severity at the onset of the FN episode. In three contemporary series, the frequency of unexpected serious complications in apparently stable individuals in the first 3 h was 7.3–18.6% [25, 29]. The Clinical Index of Stable Febrile Neutropenia (CISNE) (Table 2) was developed and validated for the Western population to predict serious complications (shock, acute organ failure, arrhythmia, major bleeding, delirium, disseminated intravascular coagulation, acute abdomen), in the subgroup of patients with solid tumors who have undergone mild–moderate-intensity chemotherapy and who appear to be clinically stable at the onset of the episodes [II, B] [14, 25, 26, 29]. Its application requires having previously ruled out hemodynamic instability or clinical risk of complications by means of vital signs, anamnesis, physical examination, and laboratory data (Table 3). CISNE should never be used in hematological patients or individuals with lymphoma, those who are unstable, with serious infections, or who have contraindications for ambulatory treatment for other reasons. There is no experience regarding its application to select patients at low risk for direct ambulatory treatment. Online calculators for CISNE are available and provide a categorized (https://www.mdcalc.com/clinical-index-stable-febrile-neutropenia-cisne) or continuous estimate of risk (http://www.iricom.es/prognostictools/cisne/inicio.aspx).

**Table 3:** The CISNE score

Characteristics	Weight (points)
ECOG-PS ≥ 2	2
Stress-induced hyperglycemia	2
Chronic obstructive pulmonary disease	1
Chronic cardiovascular disease	1
Mucositis NCI grade ≥ 2	1
Monocytes < 200 per μL	1

CISNE prognostic categories	Score^a^
Low risk	0
Intermediate risk	1–2
High risk	3–8

^a^A CISNE score ≥ 3, equals ≥ 116 points on the nomogram, or a risk of ≥ 13%, on the online calculator

The usefulness of the *Multinational Association of Supportive Care in Cancer* (MASCC) is unknown in predicting complications in patients deemed stable and one must be cognizant of its methodological limitations (coincidence of hypotension as the main predictor and endpoint, heterogeneous population with hematological neoplasms, etc.) [1], and of its low sensitivity in stable patients (34.8–36%) [23, 24, 26, 31–34].

## Initial empirical treatment

Empirical treatment should be selected on the basis of the type and severity of the infection, and the probability of antibiotic-resistant microorganisms being involved in its etiology. The first dose of empirical antibiotic therapy should be initiated within the first hour and after taking samples for culture; hospitals should, therefore, adopt suitable logistical measures [II, A].

For all patients, intravenous administration of a β-lactam with antipseudomonal activity but that conserves activity against Gram positive is recommended (piperacillin–tazobactam 4.5 g/6 h; meropenem 1 g/8 h; imipenem–cilastatin 500 mg/6 h; cefepime 2 g/8 h) [I, A]. This regimen must be modified depending on the patient’s prognosis and characteristics, as indicated below:

- **High-risk patients**: parenteral β-lactam with antipseudomonal activity should be combined with another agent chosen on the basis of the clinical situation:
  In the event that piperacillin–tazobactam is used, amikacin 15–20 mg/kg/day intravenous [IV] should be associated, or substitute the β-lactam for imipenem or meropenem due to the growing problem of *Pseudomonas* spp. infections, particularly in oncological patients, and ESBL-producing Gram-negative bacilli [II, A].
  In situations of severity (e.g., quick SOFA ≥ 2 points), associate amikacin 15–20 mg/kg/day IV (higher doses may be appropriate depending on isolation and pharmacokinetic data) [II, A] to the β-lactam (preferably a carbapenem).
  When a catheter-related infection, skin focus, or pneumonia is suspected or if the patient presents hemodynamic instability, associate vancomycin (15–20 mg/kg/8–12 h IV), linezolid (of choice if the focus is pulmonary or cutaneous, but not recommended in catheter-related infections), or daptomycin (of choice in severe patients with quick SOFA ≥ 2 points and suspicion of cutaneous or catheter focus) to the β-lactam [II, A]. Tigecycline (50–100 mg/12 h IV after an initial dose of 100–200 mg) is an alternative that may be used as a last option given the increase in all-cause mortality observed in a meta-analysis.
  In case of enterocolitis (typhlitis) or perirrectal infection, the previously mentioned β-lactams are active; however, given the risk of possible resistance, the recommendation is that parenteral metronidazole 500 mg/6 h be associated [II, A].
  In the event of prior carbapenem-resistant *Pseudomonas aeruginosa* colonization or of high risk due to high incidence of said strains in the patient’s setting and severity, assess the use of ceftolozane–tazobactam or ceftazidime–avibactam (unless resistance is already known) as β-lactam or associate colistimethate.
- In **patients who are allergic to penicillin**, the β-lactam must be substituted and aztreonam 1 g/8 h in association with vancomycin (or linezolid, tigecycline or daptomycin; see previous paragraph) used in its place, assessing the addition of metronidazole and/or amikacin, according to previous indications [II, B]. Consider the possibility that *Pseudomonas aeruginosa* may be resistant to aztreonam; therefore, in the previously mentioned situations it is wise to always associate amikacin, and later, with the data from the antibiogram in hand, assess the patient’s desensitization to the β-lactam of choice according to the microbiology data and focus of infection [II, B].
- **Low-risk patients** for infection by resistant microorganisms and for complications: after the first intravenous doses, if the patient has not received prophylaxis with fluoroquinolones, oral treatment (always in combination) is an alternative that is deemed effective:
  Amoxicillin–clavulanic 875/175 mg/8 h or 1000/62.5 mg 2 comp/12 h (*per os*) (clindamycin 300 mg/8 h in case of allergy to penicillin) + Levofloxacin 750 mg/day P.O. or ciprofloxacin 750 mg/12 h P.O. [II, B]. In patients who are allergic to β-lactams, there is no good oral treatment option. In these cases, the initial treatment should be intravenous; hospital discharge is not recommended until microbiological documentation is available or after 48–72 h of clinical stability.

In our setting, in which enterobacteria resistant to quinolones and non-fermenting bacteria are high (up to 50%), initial treatment with fluoroquinolones in monotherapy is not recommended, not even in low-risk patients [II, D]. In general, antibiotic doses must be adjusted whenever necessary for the patient’s kidney function.

Therapeutic use of granulocyte-colony stimulating factor (G-CSF) is recommended in episodes of FN in patients at high risk for infectious complications, with profound neutropenia (< 100 neutrophils/mm3), or in the presence of adverse prognostic factors, such as age > 65 years, unstable patients, with serious complications or widespread infections [I, A] [35, 36]. Their routine use is not recommended, since, although it decreases duration and hospital stay, it does not enhance survival [35, 36].

## Treatment in special situations

For infections due to certain resistant microorganisms or in special situations, there are specific recommendations to modify the general regimen, collected in Table 4.

**Table 4:** Treatment in special situations (consultation with an infectious disease specialist is recommended)

Situation	Diagnostic/treatment recommendation	Level of evidence and recommendation
ESBL-producing enterobacteria	Treatment with carbapenems	III, A
Multi-resistant *Acinetobacter* spp. in severe patients and those with pulmonary symptoms	Associate inhaled colistimethate (2 MU/8 h) and parenteral: loading dose 6–9 MU IV followed by 2–3 MU/8 h	III, B
*Stenotrophomonas maltophilia*, especially in patients with CVC or with pulmonary symptoms or infiltrates	Add cotrimoxazole 5 mg/kg/6 h IV	III, B
*Pneumocystis jirovecii* or in patients with interstitial infiltrated associated with marked lymphopenia, prolonged use of steroids or biological treatments (anti-tumor necrosis factor (TNF) monoclonal antibodies, or anti-lymphocytes), if associated respiratory symptoms	Cotrimoxazole 5 mg/kg/6 h IV, add prednisone 40 mg/12 h IV in patients with pO2 < 70 mmHg and perform bronchoscopy or induced sputum for microbiological study	II, B–C
Previous *Clostridium difficile* or in patients with diarrhea, after stool sampling	Vancomycin 250–500 mg/6 h *per os* as first choice and associated with metronidazole 500 mg/8 h IV in high-risk patients. In case of oral intolerance, the vancomycin can be administered by NGT or by means of enemas	I, A
Methicillin-resistant *Staphylococcus aureus*	In general, add vancomycin or linezolid (the latter is the drug of choice in the event of a pulmonary focus or skin and soft tissue infection)	I, A
	In patients with primary MRSA bacteremia or skin focus and criteria of severity, in the absence of pneumonia, consider the switch to daptomycin (6–8 mg/kg/day IV)	II, B–C
Suspected or confirmed Gram-positive bacteremia related with central venous catheter, in particular if the central line cannot be removed and the patient’s clinical situation is serious	Add vancomycin	II, A
	Linezolid is not recommended	III, D
	In patients meeting severity criterion or persistence of bacteremia, consider switching to daptomycin and consulting with a specialist in infectious disease for combined treatments	II, B
*Enterococcus faecalis*	In our setting, the treatment of choice is ampicillin 2 g/4–6 h IV. It is important to document negativization of bacteremia	III, A
*Enterococcus faecium* and ampicillin-resistant species	Vancomycin, linezolid, or daptomycin depending on the focus of infection	
Carbapenemase-producing *Enterobacteriaceae*	Ceftazidime/avibactam (KPC-2 or OXA-48-producing strains). In case of infection due to carbapenemase-producing or carbapenem-resistant strains due to the loss of porins together with hyper-production of Amp-C, associations of two or three of the following antibiotics: colistin, tigecycline, fosfomycin (if the strain is sensitive), an aminoglycoside (amikacin or gentamicin) and meropenem (if the MIC ≤ 16 mg/L), administered in high doses and continuous perfusion	III, A
Symptoms suggestive of flu during the winter flu season peak	Samples should be taken for study by nucleic acid amplification techniques (NAATs) of the flu virus. Empirical treatment should be administered with oseltamivir 75 mg/12 h P.O.	II, A
Documented respiratory syncytial viral infection in patients with marked lymphopenia	The therapy with ribavirin may be beneficial for some high-risk patients	III, C
Symptoms and lesions clinically compatible with chicken pox or herpes zoster	Empirical treatment with acyclovir (10 mg/kg/8 h iv)	I, A
Patients receiving prolonged, high-dose steroids or TNF antagonists in the presence of suggestive symptoms (prolonged fever without a focus, interstitial pneumonia, nephritis, hepatitis, cholangitis, esophagitis, colitis, pancreatitis, encephalitis, or uveitis)	Diagnostic studies for the detection of CMV; initiate treatment with ganciclovir if the result is positive or in case of clinical worsening with a high degree of suspicion	II, A

As regards fungal infections, empirical antifungal treatment must be initially considered (“preemptive therapy”) if, after 5–7 days of antibiotic treatment, the patient continues to be febrile and the initial microbiological studies are negative [I, A]; the most appropriate time depends on the clinical setting and the presence of risk factors for invasive fungal infection (IFI) (prolonged neutropenia, immunosuppressant treatment, corticosteroids, etc.). A galactomannan titer in blood of > 0.5 (optical density index) on two consecutive determinations, or > 0.7 in a single determination, is valuable for the diagnosis of invasive aspergillosis. Furthermore, the IFI must be characterized by means of targeted studies based on clinical suspicion (CT; ear, nose and throat exploration, lung puncture aspirate, biopsy of skin lesions, etc.) [III, A]. The choice of antifungal drug (echinocandin, voriconazole, or liposomal amphotericin B) is conditioned by the prior use of azoles, clinical suspicion, and results of the galactomannan test. Echinocandins would only be of choice in cases of suspected *Candida* spp. infection (patients with widespread mucositis, patients with CVC and/or parenteral nutrition); in the event that *Aspergillus* spp. infection is suspected (lung nodules, positive galactomannan test), voriconazole is the drug of choice [37]. At centers with a high incidence of Mucorales, initiating liposomal amphotericin B treatment must be evaluated.

## Hygiene–dietary considerations

Patients with FN are sometimes prescribed a diet low in bacteria, without having confirmed that this diet lowers the risk of infection or mortality [III, C] [38]. Hand hygiene is the most effective procedure for preventing hospital acquired infections; all personnel should, therefore, follow hand-washing protocols before entering and after leaving the room of a neutropenic patient, as well as the “5 moments” approach of hand-washing as per the guidelines of the World Health Organization [I, A] [39]. General protection measures should be adopted (e.g., gloves, gowns, and/or masks) [40] and the contact isolation measures established in each for specific isolations: methicillin-resistant *Staphylococcus aureus*, vancomycin-resistant *Enterococcus spp*, carbapenemase- or ESBL-producing *Enterobacteriaceae*, non-fermenting Gram-negative bacilli, such as *Pseudomonas aeruginosa* or *Acinetobacter baumannii*, with a pattern of multi-resistance [II, A]. Moreover, patients should optimize their oral and skin hygiene [II, A]. In patients with solid tumors, reverse isolation is indicated only for patients being treated with chemotherapy schedules that produce profound and prolonged neutropenia [II, A] [40].

## Follow-up and treatment adjustment

During follow-up of a patient with FN, a physical examination must be performed and their general daily status must be evaluated to rule out the appearance of new infectious foci. Vital signs must be taken every 8 h. Hemogram and biochemistry with daily kidney and liver function tests must be carried out, and blood cultures every 48 h in patients with persistent fever, in addition to sampling of other foci that may appear. Supervision, prevention, and treatment of other complications (thrombosis, anemia, thrombopenia, intestinal dysbacteriosis, mucositis, etc.) are needed [III, A].

The adjustments made to the antibiotic regimen will depend on the patient’s evolution, the infectious focus, and the sensitivity profile of the microbiological isolations. Likewise, if the fever persists and the focus of infection is identified, the antibiotherapy should be modified based on a specific suspicion or on the microbiological isolation [I, A]. In the case of having initiated specific empirical treatment against Gram-positive bacilli (e.g., glycopeptide), it must be withdrawn 48 h after its initiation if the initial suspicion is not confirmed [II, B]. In patients who have been febrile > 48 h without clinical worsening, a wait and see approach can be adopted, actively searching for the clinical source of infection by means of serial blood cultures, stool cultures, and imaging studies depending on the clinical suspicion. Similarly, peripheral venous catheters should also be rotated periodically and non-infectious causes of fever should also be assessed. In patients with persistent fever > 48 h who also present hemodynamic instability or clinical progression, the empirical antimicrobial spectrum should be expanded to cover resistant Gram-negative bacilli, Gram-positive bacteria, and anaerobes [I, A]. Antifungal medications may be necessary in patients who meet criteria of severity and have persistent fever > 4–7 days (see treatment in special situations, above). In cases that so require and depending on the patient’s options for recovery and the cancer’s prognosis, the intensive care unit will be contacted [41]. The isolation of pathogens resistant to one or more of the components of the initial antibiotic therapy entails the need to adjust it (Table 4). A specialist in infectious disease or experienced clinician should be consulted when faced with patients with poor evolution, *S. aureus* bacteremia, persistent bacteremia, atypical microorganisms or resistances, highly severity infectious syndromes (CNS involvement, endocarditis, etc.), the need for antimicrobials with which the attending oncologist is relatively unfamiliar (Table 3), or HIV infection [III, C].

Empirical antibiotic treatment should last for a minimum of 7 days in low-risk FN without an identified clinical or microbiological focal site. When the neutrophil count exceed 500 cells/mm3 and the patient has been fever free for 72 h, oral administration can commence (as per the same considerations as in the previous section) [III, A]. In FN with clinical or microbiologically documented infection, said treatment should be followed in accordance with the recommended duration for the underlying infectious process [III, B]. In the specific case of skin and soft tissue infections, pneumonias, and urinary tract infections, antibiotic treatment should be followed for 10–14 days in most cases [II, B]. In the event of microbiological documentation, the antibiotic spectrum can be decreased depending on the focus and severity of the infection, and the antibiogram of the microorganisms identified as the cause of the infectious disease [II, A].

## Inpatient treatment, early discharge, and outpatient treatment

Hospital treatment should be considered standard, whereas home treatment or early discharge is an alternative in selected, low-risk cases [4], defined as those that have no severity criteria [II, A] (Table 2). The CISNE model should only be used in patients in a situation of apparent clinical stability, after having ruled out clinical, epidemiological, or analytic criteria of severity, with the aim of avoiding early discharge of patients at potential risk for complications or severe sepsis.

**Fig. 1:**
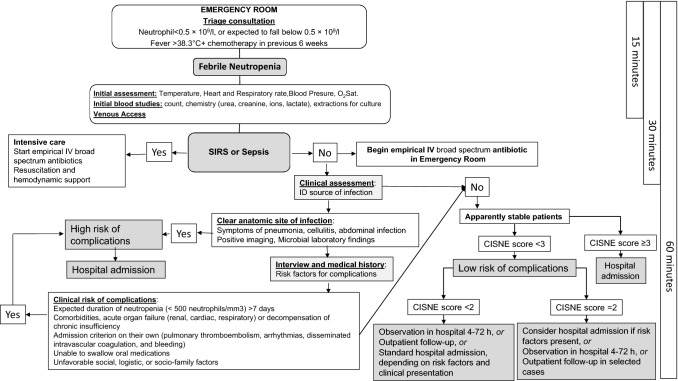
Decision-making algorithm in febrile neutropenia

CISNE does not seek to select patients for outpatient management, but to safeguard them from inappropriate discharge until favorable evolution is confirmed. Figure 1 illustrates the decision-making algorithms that make up the CISNE score.

In particular, stable patients who are qualified as high risk according to CISNE (score ≥ 3, its equivalent ≥ 116 points on the nomogram, or risk for complications ≥ 13% on the on-line calculator) should be treated in the hospital and should not be discharged until observation has demonstrated that the apparent stability is real and blood cultures have proven negative [II, B] [26, 29, 42]. In patients with a CISNE score of 2, additional clinical or microbiological risk factors should be considered that might also contradict ambulatory management.

In patients who are at low risk (score CISNE 0–1), admission to hospital with early discharge, standard hospital admission or ambulatory treatment with close follow-up can be considered, depending on each episode’s clinical presentation [III, B]. In any case, given the potential risk of unexpected complications even in the lowest risk group (4.2% of the patients with 0–57 points on the CISNE nomogram) [29], for a patient with FN to be treated at home, all the following criteria must also be met: reside within 1 h or less than 50 km from the hospital; have a means of transportation available, access to a telephone, and good family support in the home for 24 h; have the approval of their referring oncologist/clinician; foresee good compliance with regular doctor’s visits; expected compliance with the oral administration of medications; and no prior history of non-compliance with treatment [III, B] [32]. After being identified as low risk and initiating empirical antibiotic therapy and prior to discharge, patients must remain in observation for at least 4 h to verify their stability and tolerance to treatment [III, C] [32]. If the decision is made to discharge the patient, close follow-up is mandatory with clinical reevaluation and hemogram 48 h later, and daily phone contact for a minimum of 3 days to check that the fever has resolved. It is important to properly inform the patient (orally and in writing) about the detection of warning signs and symptoms, as well as how to contact the healthcare personnel 24 h/day [III, B] [29, 32, 43]. Hospital admission must be contemplated when fever or neutropenia persists after 2–3 days of treatment with empirical antibiotic therapy, new signs or symptoms of infection, oral intolerance, or need to change antibiotherapy [III, B] [32].

## Prevention of febrile neutropenia: prophylaxis with G-CSF

The risk of developing FN must be evaluated on a case-by-case basis at the beginning of each cycle of chemotherapy [44]. Primary prophylaxis with G-CSF is recommended when the likelihood of developing FN is > 20% [2, 11, 45, 46], having demonstrated a decrease in incidence, duration, and severity, without significantly affecting tumor response or survival [I, A] [35, 47]. In patients with an intermediate level of risk (10–20%), the indication of prophylaxis with G-CSF must be individualized depending on the characteristics that increase the risk of FN (age > 65 years, liver or kidney dysfunction, widespread bone marrow involvement, recent extensive surgery, persistent neutropenia, or prior episodes of FN) [II, A] [2, 11, 45, 46]. In special circumstances, regardless of frequency, individuals may also be considered eligible for primary prophylaxis if the consequences of the neutropenic episode are foreseen to be more severe.

The recommended dose of filgastrim is 5 µg/kg/day subcutaneously administered 24–72 h after administration of chemotherapy until achieving a sufficient, stable post-nadir neutrophil count [I, A]. Pegfilgastrim (a single dose of 100 µg/kg or 6 mg) can be more effective in different contexts and approved biosimilar drugs can also be considered [2, 45].

In patients who have experienced a prior episode of FN or dose-limiting neutropenia [48], the use of secondary prophylaxis with G-CSF can be considered individually if the dose reductions or delay of antineoplastic treatments can negatively affect prognosis [I, A] [2, 11, 45, 46].

## Prevention of febrile neutropenia: antimicrobials and vaccination

In patients with solid tumors treated with chemotherapy of mild–moderate intensity, the use of prophylaxis with oral antibiotics to prevent episodes of FN [I, E] is not recommended, except for exceptional cases of patients with very high risk of infection in whom antineoplastic therapy cannot be delayed [III, B].

In patients with specific risks, prophylaxis against *Pneumocystis jirovecii*, tuberculosis [49], or viral hepatitis should be assessed according to recommendations found in other clinical practice guidelines [II, A].

Patients with active solid tumors and those receiving chemotherapy should be vaccinated every year against flu [II, A] and against pneumococcus [III, A] in accordance with guidelines for immunodepressed patients. Other vaccinations may be advisable depending on the kind of chemotherapy, the patient’s clinical status, or a specific indication [43].

## Abbreviations

ECOG-PS: Eastern Cooperative Oncology Group performance status
NCI: National Cancer Institute
COPD: Chronic obstructive pulmonary disease
